# Phytosanitary Monitoring of Major Lepidopteran Pest and Their Natural Enemies in Maize Agroecosystems of Southeastern Kazakhstan

**DOI:** 10.3390/insects17101033

**Published:** 2026-10-09

**Authors:** Ainura Adilkhankyzy, Karlygash A. Alpysbayeva, Khamitzhan M. Tleubergenov, Bauyrzhan B. Nurmanov, Balzhan Zh. Naimanova, Nurgul Z. Shissenbayeva, Aidana B. Agabek, Nurzhan Tashigul, Assel M. Seitzhan, Bakhytzhan A. Duissembekov, Gulzhan Kaldybekkyzy

**Affiliations:** 1 Department of Horticulture, Plant Protection and Quarantine, Faculty of Agrobiology, Kazakh National Agrarian Research University, Almaty 050010, Kazakhstan; adilhan_ainura@mail.ru; 2 Department of Biological Plant Protection, Kazakh Research Institute of Plant Protection and Quarantine Named After Zh. Zhiyembayev, Almaty 050070, Kazakhstan; zhan280197@gmail.com (K.M.T.); bauka_92kzs@mail.ru (B.B.N.); baljan-sun93@mail.ru (B.Z.N.); shisenbaevan00@gmail.com (N.Z.S.); aidanaagabek25041995@gmail.com (A.B.A.); nurzhan9601@mail.ru (N.T.); asselseitzhan@mail.ru (A.M.S.); bduisembekov@mail.ru (B.A.D.); gkaldybekkyzy@bk.ru (G.K.); 3 Department of Technique and Technology of Food Production, International Engineering and Technological University, Almaty 050060, Kazakhstan

**Keywords:** *Zea mays*, *Ostrinia* spp., *Helicoverpa armigera*, pheromone monitoring, seasonal dynamics, natural enemies, parasitoids, predators, biological control, Kazakhstan

## Abstract

Information on the seasonal dynamics of major maize pests and naturally occurring parasitoids and predators in agroecosystems of southeastern Kazakhstan remains limited. Regional data on the seasonal occurrence of phytophagous insects and their natural enemies are needed to improve phytosanitary monitoring and support the development of environmentally oriented approaches to maize protection. The study was conducted from 2022 to 2025 in commercial maize fields in the Almaty and Zhetysu regions of southeastern Kazakhstan. Phytosanitary surveys included visual monitoring of pests and their developmental stages on selected plants, assessment of overwintering *Ostrinia* spp. populations in post-harvest maize residues, pheromone monitoring of the seasonal activity of *Ostrinia* spp. and *Helicoverpa armigera*, and recording of naturally occurring parasitoids and predators. The major lepidopteran pests recorded were *Ostrinia* spp. and *H. armigera*. Pheromone monitoring revealed year-to-year differences in the timing and magnitude of seasonal trap catches of both taxa. For *Ostrinia* spp., several periods of increased trap catches were recorded during the growing season, primarily from June to August, with the highest mean catch of 23 males per trap recorded in the third ten-day period of June 2024. For *H. armigera*, the timing of maximum trap catches varied among years, with the highest mean catch of 23 males per trap recorded in the first ten-day period of August 2025. Viable overwintering larvae of *Ostrinia* spp. were found in all 11 surveyed farms. Maize residues also contained larvae parasitized by Braconidae, tachinid pupae, and individuals showing signs of bacterial infection. During the growing season, representatives of Braconidae, Trichogrammatidae, Chrysopidae, Coccinellidae, and Tachinidae were recorded in maize agroecosystems. The findings provide a regional baseline for improving phytosanitary monitoring of maize in southeastern Kazakhstan. The observed year-to-year differences in seasonal trap catches highlight the importance of accounting for variation among individual growing seasons, while the naturally occurring parasitoids and predators recorded in this study warrant further investigation to assess their potential role in environmentally oriented maize pest management programs.

## Simple Summary

Maize is an important crop in Kazakhstan, but insect pests can reduce crop yield and quality. In southeastern Kazakhstan, information about when the main maize pests appear, how their numbers change during the growing season, and which naturally occurring insects can help control them is still limited. In this study, we examined commercial maize fields in the Almaty and Zhetisu Regions and monitored the main insect pests and their natural enemies. We found that *Ostrinia* spp. and the cotton bollworm *Helicoverpa armigera* were the main lepidopteran pests recorded in the surveyed maize fields. Their activity varied considerably between years, showing that regular monitoring is important for timely pest management. Increased pheromone-trap catches of *Ostrinia* spp. were recorded at several points during the growing season, primarily from June to August. We also recorded several groups of naturally occurring parasitoids and predators, including parasitoid wasps, lacewings, lady beetles, and tachinid flies. These natural enemies represent components of the maize agroecosystem and warrant further investigation to determine their potential contribution to biological and integrated pest management in Kazakhstan.

## 1. Introduction

Maize (*Zea mays* L.) is one of the most important crops worldwide and ranks first among cereal crops in terms of global production. According to the Food and Agriculture Organization of the United Nations (FAO), annual global maize production exceeds 1.2 billion tonnes, and its importance continues to grow due to rising demand for food, animal feed, and industrial raw materials [1,2]. In Kazakhstan, maize is considered a strategically important crop used for grain, silage, and seed production. The main maize-growing areas are concentrated in the southeastern regions of the country, where favorable agroclimatic conditions provide high yield potential and create opportunities for further expansion of maize production.

At the same time, intensifying maize production increases phytosanitary pressure on agroecosystems. The expansion of monoculture, changing climatic conditions, and increasing accumulated degree-days may promote pest population growth, alter their seasonal dynamics, and facilitate range expansion. Among the most economically important pests of maize are lepidopteran species, particularly representatives of the genus *Ostrinia*, whose larvae damage stems, leaves, tassels, and ears, and the cotton bollworm, *Helicoverpa armigera* (Hübner), whose larvae feed on the reproductive structures of maize and may cause substantial yield losses. In addition to direct feeding damage, injuries caused by these pests can facilitate the entry of phytopathogenic microorganisms and contribute to deterioration in crop quality [3,4,5,6].

Current approaches to sustainable agriculture emphasize reducing dependence on synthetic chemical pesticides and increasing the use of natural mechanisms regulating pest populations. Natural biological control provided by communities of parasitoids and predators represents an important ecosystem service in agricultural systems. The conservation of beneficial insect biodiversity can contribute to pest suppression, reduce pesticide inputs, and enhance the ecological resilience of agroecosystems. Biological control is therefore regarded as a key component of integrated pest management and sustainable agroecosystem management [7,8,9,10,11].

In maize agroecosystems, natural pest suppression is provided by a diverse assemblage of specialized and generalist natural enemies. Among parasitoids, *Bracon* spp. and *Trichogramma* spp. are of particular practical importance, attacking lepidopteran larvae and eggs, respectively. Predatory natural enemies, including lacewings (Chrysopidae) and lady beetles (Coccinellidae), also contribute to pest suppression, while tachinid flies (Tachinidae) represent another important group of parasitoids associated with lepidopteran hosts. In many countries, the contribution of these natural enemies to pest regulation has been demonstrated both under natural field conditions and within biological control and integrated pest management programs in maize. In recent years, research has increasingly expanded beyond assessments of individual biological control agents to encompass the diversity, community composition, and functional roles of natural enemies as important components of agroecosystem functioning and ecosystem service provision [12,13,14,15,16].

Despite the economic importance of maize to agriculture in Kazakhstan, recent studies providing comprehensive assessments of the phytosanitary status of maize agroecosystems remain limited. Most published studies have addressed specific aspects of the biology of individual pest species or evaluated the efficacy of particular plant protection methods. However, information on the composition of the major phytophagous insect fauna and their associated natural enemies, as well as their seasonal occurrence in maize agroecosystems of southeastern Kazakhstan, remains scarce. Furthermore, existing information on the species composition of maize-infesting *Ostrinia* in the region is largely based on historical literature records. Molecular identification of the local population is currently ongoing; therefore, in the present study, field-collected specimens and observed populations are referred to as *Ostrinia* spp. without definitive assignment to a particular species.

We hypothesized that maize agroecosystems in southeastern Kazakhstan support recurrent populations of major lepidopteran pests together with an assemblage of naturally occurring parasitoids and predators, and that the seasonal activity of the major pest taxa varies among growing seasons. Accordingly, the aim of this study was to assess the phytosanitary status of maize agroecosystems in southeastern Kazakhstan, characterize the seasonal activity of the major lepidopteran pests, and document the taxonomic composition and occurrence of naturally occurring parasitoids and predators associated with these pests.

## 2. Materials and Methods

### 2.1. Study Area

The study was conducted from 2022 to 2025 in two major maize-growing regions of southeastern Kazakhstan: Panfilov District in the Zhetysu Region and Enbekshikazakh District in the Almaty Region.

**Figure 1:**
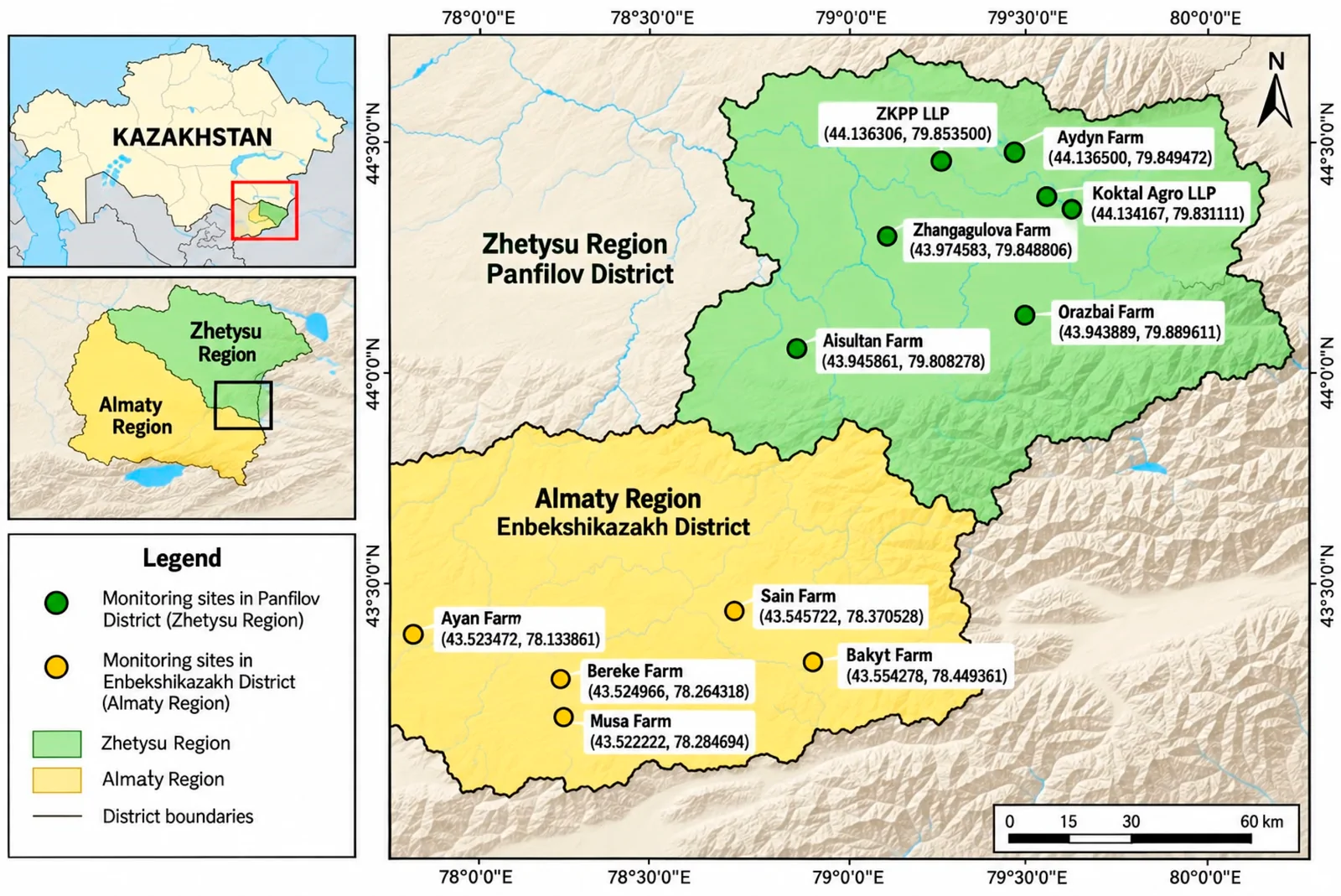
Geographic locations of the surveyed maize fields in southeastern Kazakhstan, 2022–2025. Note: Coordinates are provided in the WGS 84 coordinate reference system (EPSG:4326).

A total of 11 commercial maize fields were included in the study. In Panfilov District, six fields were surveyed: ZhKPZ LLP (300 ha), Orazbai farm (10 ha), Zhantagulova farm (100 ha), Aisultan farm (15 ha), Aiydyn farm (200 ha), and Koktal Agro LLP (300 ha). In Enbekshikazakh District, five fields were surveyed: Ayan farm (34 ha), Musa farm (5 ha), Bereke farm (7 ha), Bakyt farm (7 ha), and Sain farm (100 ha). The geographic locations and coordinates of the surveyed fields are shown in Figure 1.

Pioneer maize hybrids were grown in all surveyed fields. According to information provided by the farm owners, the crops were treated twice with chemical plant protection products during the growing season.

### 2.2. Assessment of Overwintering Populations of Ostrinia spp.

From 2022 to 2025, overwintering populations of *Ostrinia* spp. were assessed in commercial maize fields located in Panfilov District of the Zhetisu Region and Enbekshikazakh District of the Almaty Region. Surveys were conducted after harvest and before the beginning of the subsequent growing season by collecting maize crop residues for subsequent laboratory examination.

Plant material was collected using a route-based sampling approach. In each surveyed field, 10 sampling points were established, and 10 maize stalks were collected at each point, resulting in a total sample of 100 stalks per field. Different commercial fields were surveyed in different years. In 2022, two fields were surveyed (ZhKPZ LLP and Orazbai farm); in 2023, four fields (Zhantagulova, Sain, Musa, and Bakyt farms); in 2024, three fields (Aisultan, Ayan, and Aiydyn farms); and in 2025, two fields (Koktal Agro LLP and Bereke farm). Overall, 11 fields were surveyed during the study period, and a total of 1100 maize stalks were collected for laboratory examination.

The collected plant material was transported to the laboratory, where the stalks were dissected and examined for the presence of overwintering stages of *Ostrinia* spp., in accordance with standard procedures used for stem-boring lepidopteran pests [17,18]. For each individual detected, developmental stage and condition were recorded. Individual records were maintained separately for viable larvae, larvae parasitized by Braconidae, tachinid pupae, and larvae exhibiting signs of bacterial or fungal infection. The abundance of overwintering *Ostrinia* spp. was expressed as the number of individuals detected per 100 examined maize stalks.

### 2.3. Phytosanitary Monitoring of Pests and Natural Enemies

**Figure 2:**
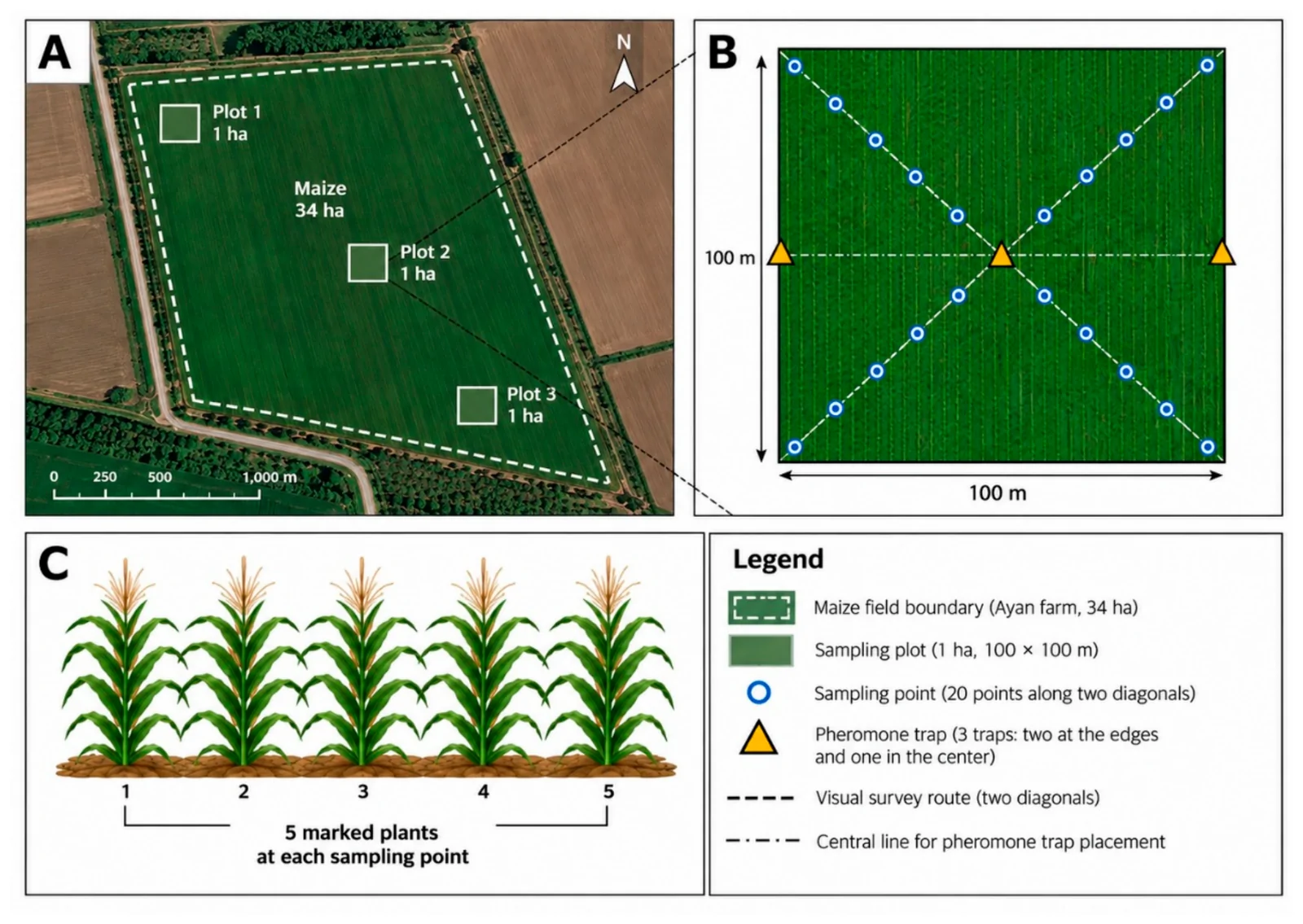
Study sites and sampling design for phytosanitary monitoring of maize: (**A**) Ayan farm, Enbekshikazakh District, Almaty Region; (**B**) Zhantagulova farm, Panfilov District, Zhetisu Region; (**C**) schematic layout of the 1-ha sampling plot showing 20 sampling points used for visual surveys and the placement of three pheromone traps.

For regular phytosanitary monitoring, two commercial maize fields were selected from the broader network of surveyed farms, representing the principal study areas: Ayan farm in Enbekshikazakh District of the Almaty Region and Zhantagulova farm in Panfilov District of the Zhetisu Region. Monitoring comprised two complementary approaches: visual plant surveys to record pests, plant injury, and naturally occurring parasitoids and predators, and pheromone-based monitoring of the major lepidopteran pests of maize. The maize field at Ayan farm covered 34 ha, whereas that at Zhantagulova farm covered 100 ha. Within each commercial field, three 1-ha sampling plots (100 × 100 m) were established (Figure 2A,B).

Visual surveys were conducted along two diagonal transects within each sampling plot. Twenty sampling points were established along the survey route in each plot (Figure 2C). At each sampling point, five maize plants were selected and permanently marked for repeated observations throughout the growing season. In the present study, these repeatedly monitored plants are referred to as marked plants. Thus, 100 marked plants were examined within each sampling plot (20 sampling points × 5 plants). Each 1-ha sampling plot was treated as the spatial observational unit, whereas individual marked plants represented subsamples within the plot. Visual assessments were conducted at 10-day intervals, with the same marked plants examined at each consecutive survey.

On each marked plant, the presence of pests, their developmental stages, and characteristic symptoms of feeding injury were recorded. Naturally occurring parasitoids and predators were recorded simultaneously. The occurrence of parasitoids was additionally verified using naturally parasitized hosts. Egg masses of *Ostrinia* spp. and larvae of lepidopteran pests showing signs of parasitism were collected in the field and transported to the laboratory. Parasitism was confirmed by the subsequent emergence of *Trichogramma* spp. from host eggs and representatives of Braconidae from host larvae. The resulting data were used to characterize the seasonal occurrence of pests and to document the naturally occurring assemblage of parasitoids and predators in maize agroecosystems.

Pheromone monitoring was conducted exclusively for the target lepidopteran pests of maize. Delta traps equipped with adhesive inserts and species-specific pheromone dispensers were used for this purpose. Pherobio dispensers (China) were used for monitoring *Ostrinia furnacalis*, whereas dispensers produced by VNIIKR (Aparikha, Russia) were used for *O. nubilalis* and *Helicoverpa armigera*.

For each pheromone dispenser type, three traps (*n* = 3) were deployed across the three 1-ha sampling plots, with one trap placed in each plot. Traps were positioned approximately 50 cm above the upper canopy level. Trap catches were assessed at 10-day intervals, and the number of males captured in each trap was recorded separately. Adhesive inserts and pheromone dispensers were replaced approximately every 20 days [19,20,21].

Catches obtained from traps baited with *O. furnacalis* pheromone dispensers were used to characterize the seasonal dynamics of *Ostrinia* spp. Adult attraction to a particular pheromone dispenser was not considered an independent criterion for species-level identification. Traps baited with *O. nubilalis* pheromone dispensers were operated in parallel; however, no adult captures were recorded in these traps during the monitoring period.

### 2.4. Insect Identification

Pests and natural enemies were identified based on morphological characteristics using specialized taxonomic keys and diagnostic guides. *Helicoverpa armigera* (Hübner) was identified based on the morphological characteristics of adults and larvae, while pheromone-trap data were used as supplementary information to characterize the seasonal activity of this species (Supplementary Table S1).

Specimens of the genus *Ostrinia* observed during visual plant surveys or captured in pheromone traps were designated as *Ostrinia* spp. in the present study because the species identity of the local population has not yet been conclusively confirmed. Adult captures in traps baited with pheromone dispensers designed for *O. furnacalis* were used to characterize the seasonal dynamics of *Ostrinia* spp.; however, attraction to the pheromone lure was not treated as a stand-alone diagnostic character for species identification.

Natural enemies were identified to the lowest taxonomic level that could be reliably determined from the available material. The groups recorded in the surveyed maize fields included *Trichogramma* spp., Braconidae, Chrysopidae, Coccinellidae, and Tachinidae.

### 2.5. Molecular Identification Methods of Bacterial Isolates

For molecular identification of the six bacterial isolates, PCR amplification was performed targeting a fragment of the *gyrB* gene, which encodes the B subunit of DNA gyrase. PCR amplification yielded products of approximately 900 bp. The amplification products were analyzed by electrophoresis in a 1% agarose gel. Selected and purified PCR products were sequenced in both directions. The resulting nucleotide sequences were compared with reference sequences available in the NCBI GenBank database using the NCBI BLAST web service.

### 2.6. Data Processing and Statistical Analysis

Data obtained from visual surveys were summarized at the level of the 1-ha sampling plot, which was considered the spatial observational unit, whereas individual marked plants within each plot were treated as subsamples. To characterize plant infestation, the proportion of plants on which a given pest or developmental stage was recorded was calculated relative to the total number of marked plants examined.

Data on overwintering populations of *Ostrinia* spp. were analyzed at the level of individual commercial fields. At each farm, 100 maize stalks were examined, and the results were expressed as the number of recorded individuals or observations per 100 examined stalks. Live larvae, larvae parasitized by Braconidae, tachinid pupae, and larvae showing signs consistent with bacterial or fungal infection were recorded separately. Because these categories included both individual insects and observations indicative of biotic interactions, the resulting values were not used to directly calculate mortality rates, parasitism rates, or the effectiveness of natural enemies.

Pheromone-monitoring data were analyzed separately for each 10-day sampling period. For each sampling date, the mean number of males captured per trap was calculated from the catches obtained from the three pheromone traps (*n* = 3):

$$\bar{x}=\frac{\sum_{i=1}^{n}x_{i}}{n},$$

where $\left(\bar{x}\right)$ is the mean number of males captured per trap during a given sampling period; ($x_{i})$ is the number of males recorded in an individual trap; and (*n*) is the number of pheromone traps.

The variability in pheromone trap catches was characterized using the standard error of the mean (SE):

$$SE=\frac{SD}{\sqrt{n}},$$

where (SD) is the standard deviation of the number of males captured among the three traps, and (*n*) is the number of pheromone traps. Pheromone-monitoring results were presented as the mean number of males captured per trap during each sampling period ± SE.

Given the hierarchical structure of the field observations, differences in the composition of the commercial fields surveyed among years, and the absence of a fully balanced repeated-measures design, the data were analyzed primarily using descriptive statistics. Interannual differences in pest abundance and timing of occurrence were considered as observed patterns of seasonal dynamics and were not interpreted as statistically significant differences among years. Similarly, data on naturally occurring parasitoids and predators obtained using different sampling methods were used to document their occurrence within maize agroecosystems rather than to quantitatively compare the relative abundance of individual natural-enemy groups or to estimate their contribution to the regulation of phytophagous pest populations.

For figures presenting pheromone-monitoring data, error bars represent the standard error of the mean calculated from the three trap replicates (*n* = 3). No inferential statistical comparisons among sampling periods or years were performed; therefore, significance-grouping letters are not presented in the figures. Data on overwintering *Ostrinia* spp. represent descriptive field-level counts per 100 examined maize stalks rather than means of replicate field samples; consequently, no error bars or inferential comparisons among farms were applied to these data.

## 3. Results

### 3.1. General Phytosanitary Characteristics of the Surveyed Maize Fields

Phytosanitary monitoring of commercial maize fields in southeastern Kazakhstan showed that maize agroecosystems were colonized by a complex of lepidopteran phytophagous insects and supported a diverse assemblage of beneficial insects, including parasitoids and predators. The major pests recorded during the study period were *Ostrinia* spp. and the cotton bollworm, *H. armigera* (Hübner).

Examination of maize crop residues during the autumn–spring period revealed the presence of overwintering stages of stem-boring pests. Viable larvae of *Ostrinia* spp. were detected in the crop residues, indicating the persistence of an overwintering pest population within the surveyed agroecosystems and its potential to initiate population development during the subsequent growing season.

During the growing season, the first signs of pest infestation were observed at the early stages of maize development. Egg masses of *Ostrinia* spp. were detected on sampled plants, predominantly on the underside of leaves. As the season progressed, characteristic damage to leaves, stems, and ears was recorded, and larvae were found inside damaged stems upon dissection.

### 3.2. Overwintering Status of Ostrinia spp.

**Figure 3:**
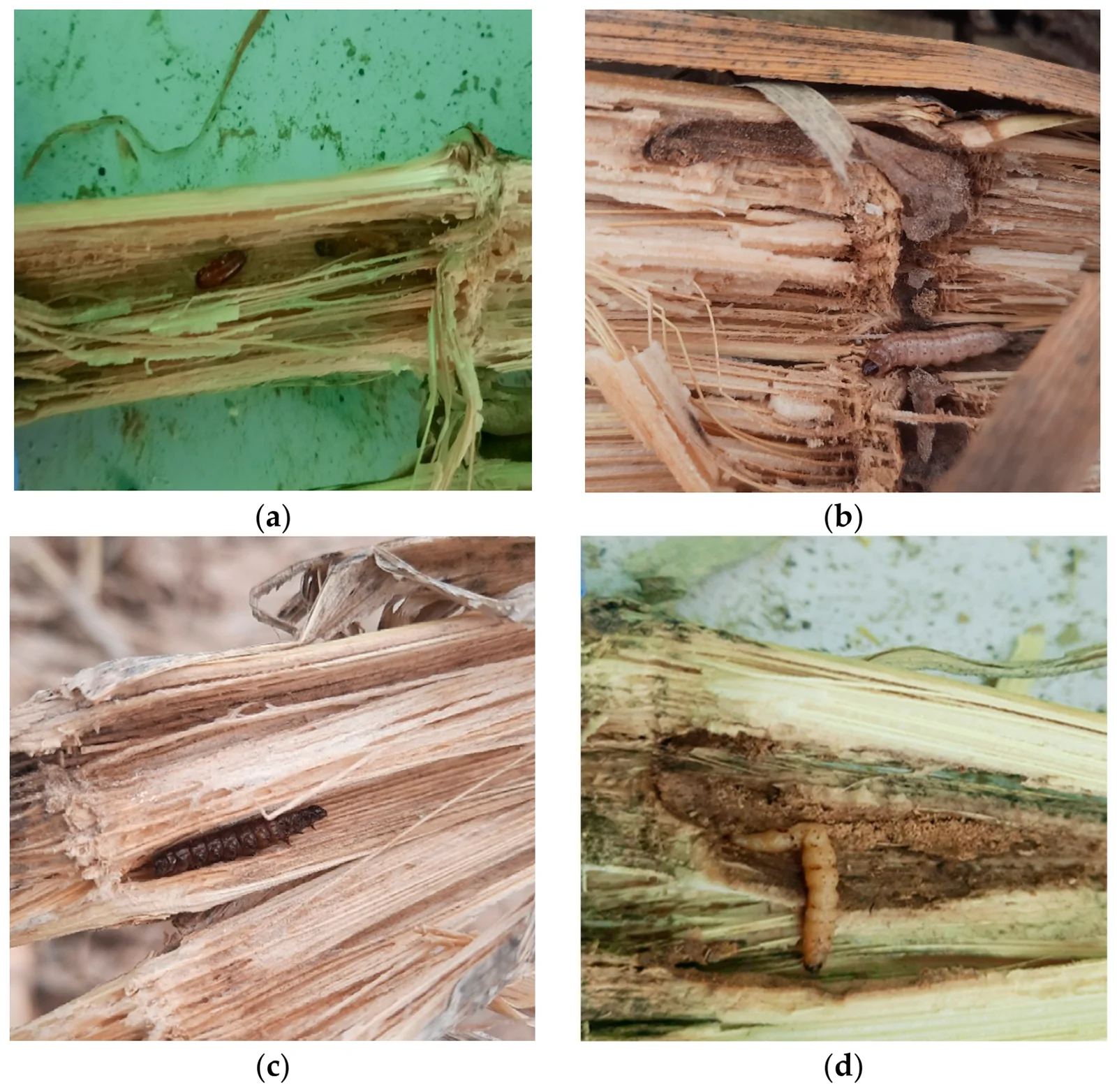
Representative observations from post-winter maize residues: (**a**) tachinid pupa; (**b**) live *Ostrinia* spp. larva; (**c**) larva showing signs of bacterial infection; and (**d**) larva parasitized by a representative of Braconidae.

Assessment of the overwintering population was conducted by examining post-harvest maize residues for viable developmental stages of *Ostrinia* spp. and for evidence of naturally occurring biological factors associated with overwintering larvae. Stem dissection revealed viable larvae as well as larvae showing evidence of parasitism or signs of bacterial or fungal infection. Representative observations are shown in Figure 3.

Quantitative assessments were conducted separately for each of the 11 surveyed farms, with 100 maize stalks examined at each farm. Viable larvae of *Ostrinia* spp. were recorded at all surveyed farms, although their abundance varied among individual fields. In the Zhetisu Region, the number of live larvae ranged from 9 to 16 individuals per 100 examined stalks, whereas in the Almaty Region, it ranged from 4 to 15 individuals per 100 stalks.

**Figure 4:**
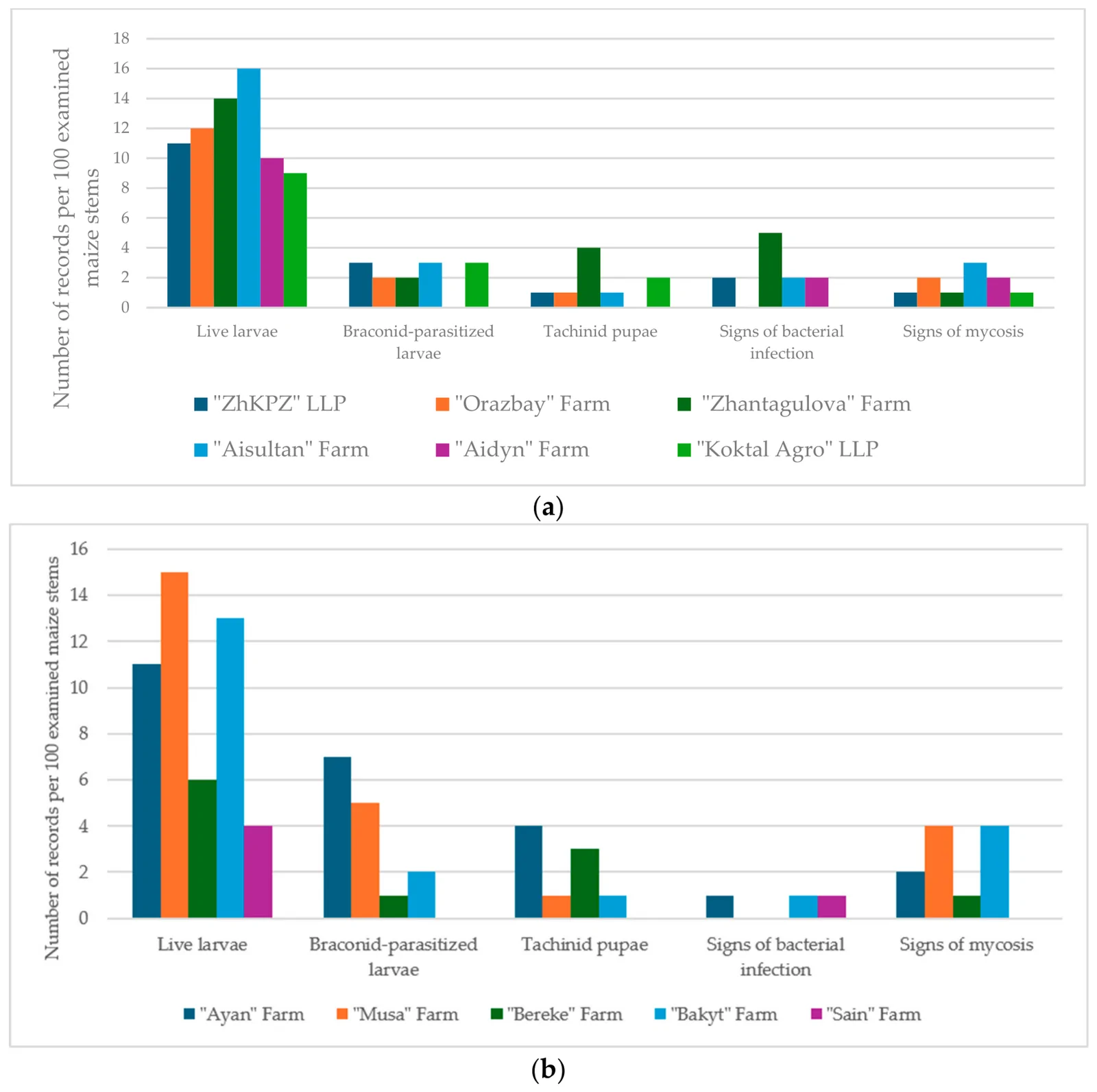
Status of overwintering *Ostrinia* spp. populations and associated biotic factors recorded in maize crop residues from surveyed farms in southeastern Kazakhstan: (**a**) Zhetisu Region; (**b**) Almaty Region. Values represent the observed number of individuals or cases per 100 examined maize stalks at each farm. These values are descriptive field-level observations rather than means of replicate samples; therefore, error bars are not applicable, and no inferential statistical comparisons among farms were performed.

Larvae parasitized by members of Braconidae were recorded in both regions, with a maximum of seven parasitized larvae per 100 examined stalks. Tachinid pupae occurred at lower frequencies and were absent from some farms. Larvae showing signs consistent with bacterial infection were recorded sporadically or at low frequencies, with a maximum of five cases per 100 stalks, whereas signs consistent with fungal infection were recorded at frequencies of up to four cases per 100 examined stalks (Figure 4).

Overall, viable overwintering larvae of *Ostrinia* spp. were present at all surveyed farms in both regions, whereas the frequency of parasitized larvae, tachinid pupae, and individuals showing signs consistent with bacterial or fungal infection varied among fields. These observations confirm the presence of naturally occurring parasitoids and individuals showing signs of possible microbial infection in maize crop residues; however, the available data do not allow direct estimation of their contribution to mortality or population regulation of overwintering *Ostrinia* spp.

### 3.3. Complex of Major Maize Phytophagous Insects

**Figure 5:**
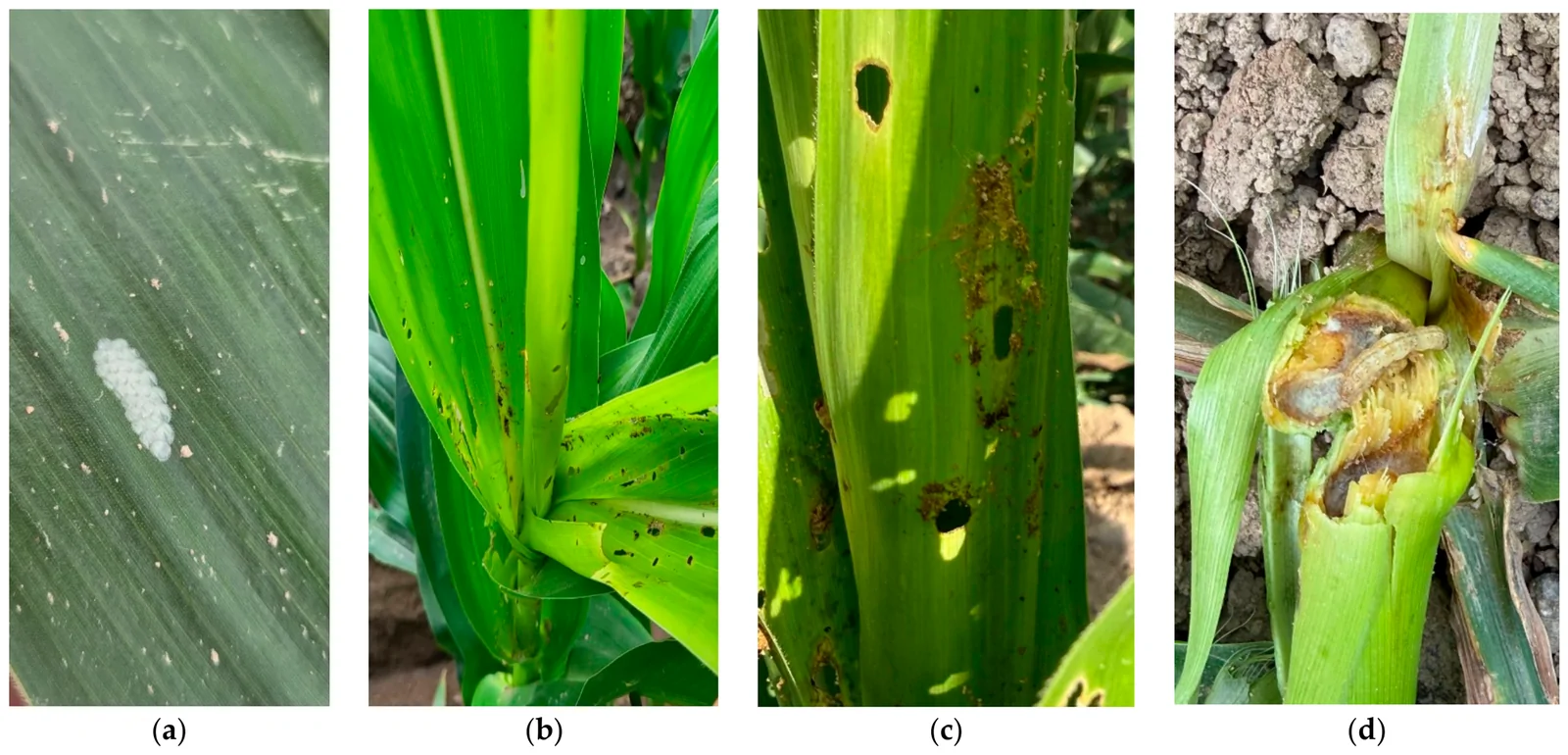
Damage caused by representatives of the genus *Ostrinia* spp.: (**a**) egg mass; (**b**) leaf damage; (**c**) entry hole; (**d**) larva inside the stem.

Field surveys showed that the principal lepidopteran pests recorded in maize fields were *Ostrinia* spp. and *Helicoverpa armigera*. Members of the genus *Ostrinia* primarily damaged maize leaves and stalks (Figure 5). During the early stages of infestation, egg masses were observed on the leaves, whereas later observations revealed feeding injury, entry holes, and damage to internal stalk tissues.

**Figure 6:**
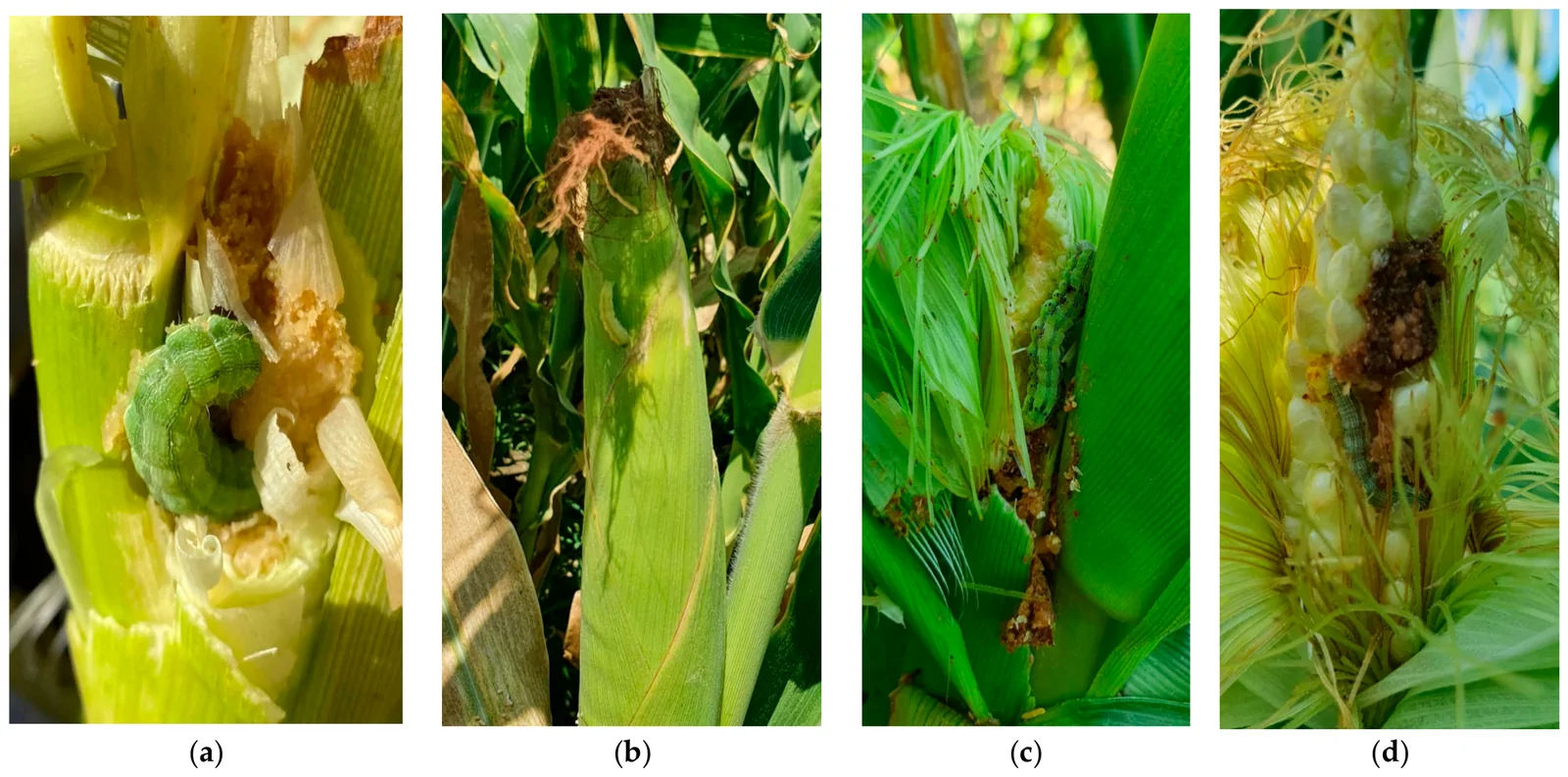
Damage caused by *Helicoverpa armigera* on maize ears: (**a**) larva feeding within the ear; (**b**) husk damage; (**c**) larva on a maize ear; (**d**) feeding damage to kernels.

*Helicoverpa armigera* was recorded primarily as an ear-feeding pest. Damage caused by this species resulted from larval feeding on the reproductive structures of maize, particularly in the ear region (Figure 6). Larvae and characteristic feeding injury were observed on damaged ears, including injury to the husks and kernels.

The phytosanitary status of the surveyed maize fields was determined by a complex of stem- and ear-feeding phytophagous insects rather than by a single pest species. Among these, *Ostrinia* spp. and *H. armigera* were the principal lepidopteran pests recorded. To further characterize the seasonal activity of the major lepidopteran pests, field survey data were complemented by pheromone-monitoring data.

### 3.4. Seasonal Dynamics of Helicoverpa armigera Trap Catches Based on Pheromone Monitoring

**Figure 7:**
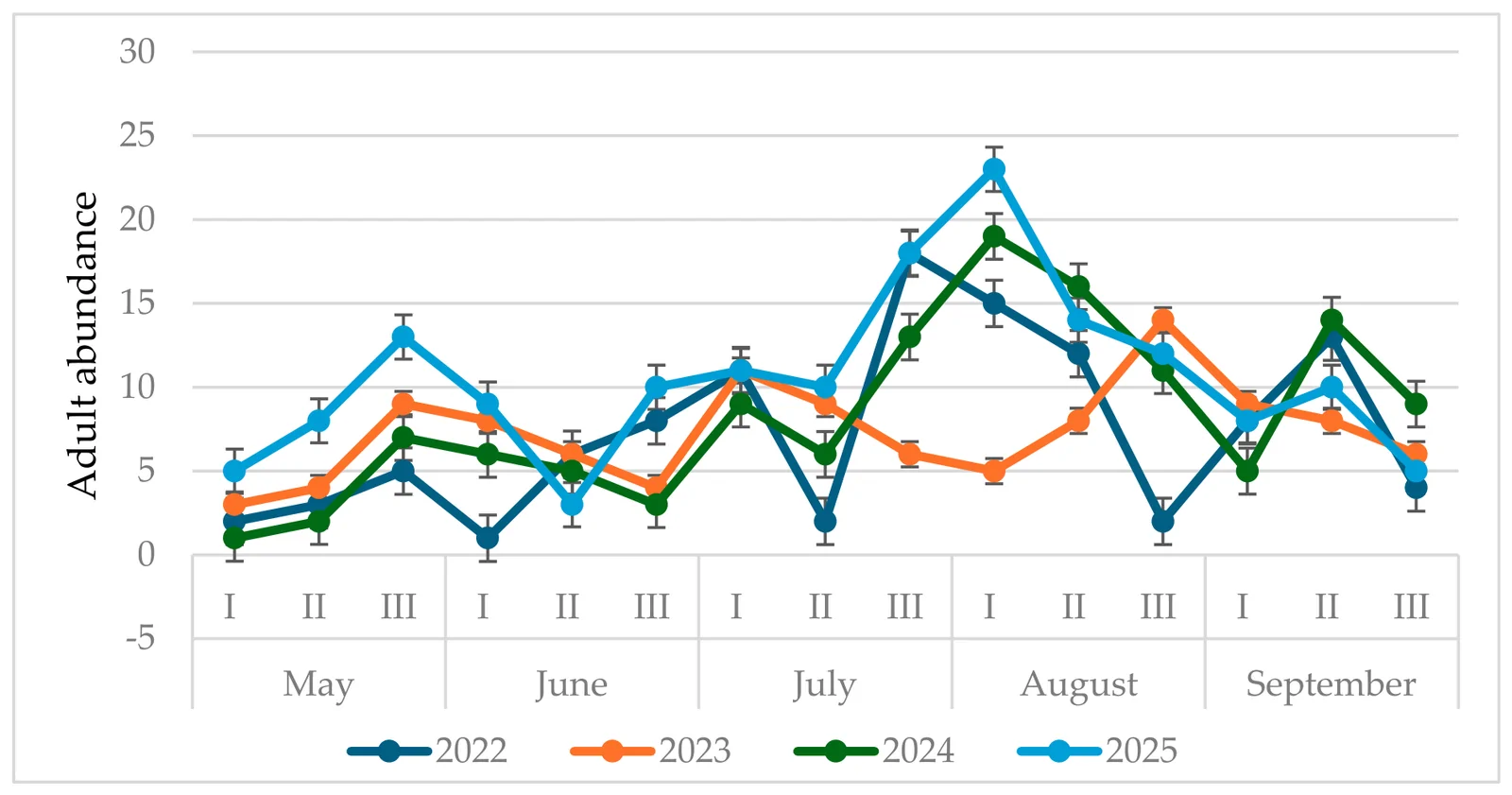
Seasonal dynamics of *Helicoverpa armigera* pheromone-trap catches in maize fields of southeastern Kazakhstan during 2022–2025. Values are presented as the mean number of males captured per trap per 10-day sampling period ± SE (*n* = 3 traps). Error bars represent the standard error of the mean (SE). I, II, and III indicate the first, second, and third 10-day periods of each month, respectively. The data are presented descriptively; no inferential statistical comparisons among sampling periods or years were performed.

Pheromone monitoring of *H. armigera* conducted from 2022 to 2025 revealed interannual variation in the seasonal dynamics of trap catches. Values are presented as the mean number of males captured per trap during each 10-day sampling period, based on three pheromone traps (*n* = 3), with the standard error of the mean (SE) shown (Figure 7).

### 3.5. Seasonal Dynamics of Ostrinia spp. Trap Catches Based on Pheromone Monitoring

**Figure 8:**
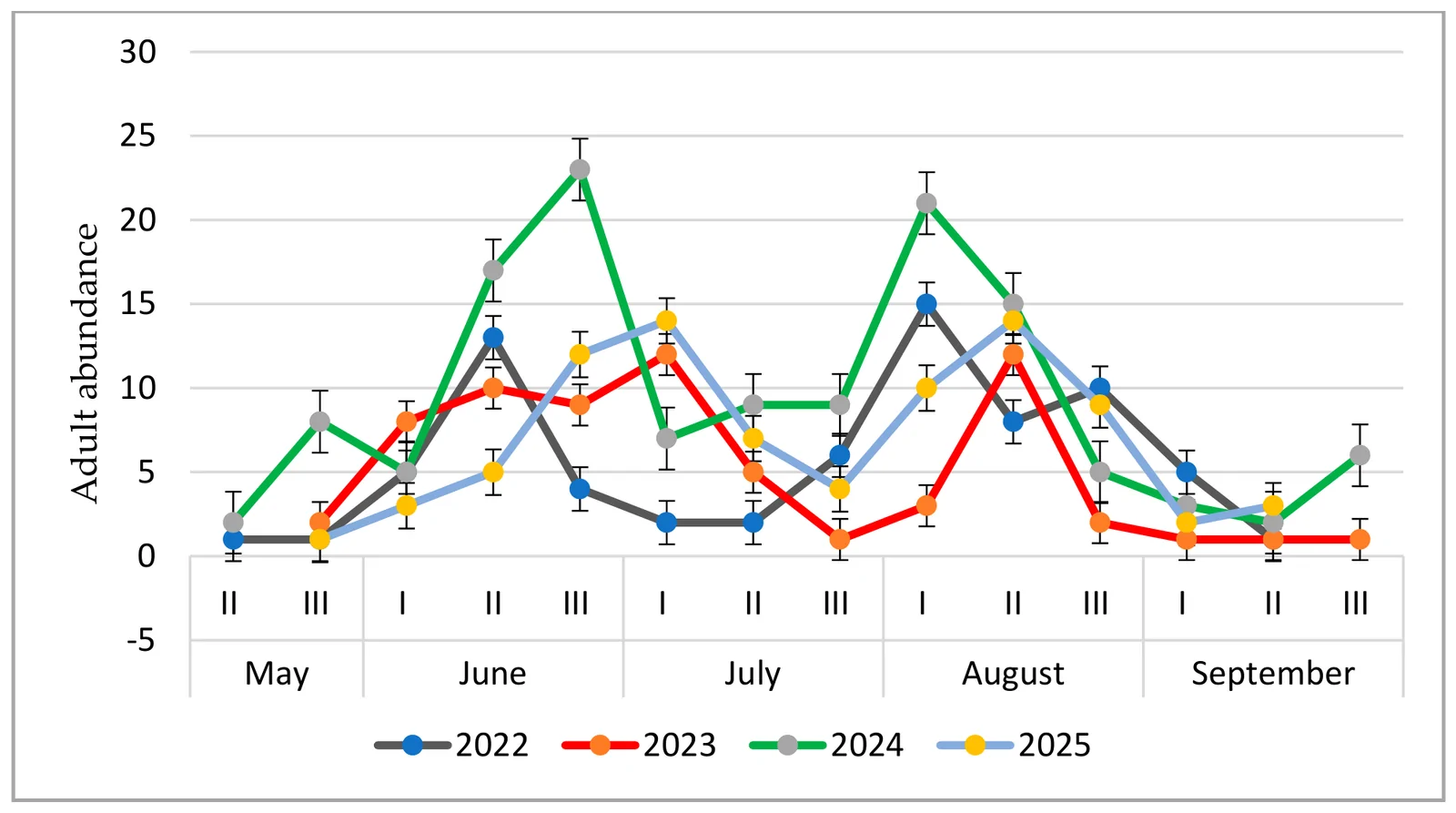
Seasonal dynamics of *Ostrinia* spp. catches in pheromone traps baited with *O. furnacalis*-specific lures in maize fields of southeastern Kazakhstan during 2022–2025. Values are presented as the mean number of males captured per trap per sampling period ± SE (*n* = 3 traps). Error bars represent the standard error of the mean (SE). I, II, and III indicate the first, second, and third 10-day periods of each month, respectively. The data are presented descriptively; no inferential statistical comparisons among sampling periods or years were performed.

Pheromone monitoring of moths of the genus *Ostrinia* was conducted from 2022 to 2025 using traps baited with pheromone dispensers designed for *O. furnacalis*. Values are presented as the mean number of males captured per trap during each 10-day sampling period, based on three pheromone traps (*n* = 3), with the standard error of the mean (SE) shown (Figure 8).

In 2022, the first pronounced increase in trap catches was observed during the second 10-day period of June, when the mean number of captured males reached 13 individuals per trap. The highest mean catch that year was recorded during the first 10-day period of August, at 15 males per trap. In 2023, the maximum mean catch was 12 males per trap and was recorded twice, during the first 10-day period of July and the second 10-day period of August. In 2024, the highest trap catches of the entire monitoring period were observed: the maximum mean catch reached 23 males per trap during the third 10-day period of June, followed by another increase to 21 males per trap during the first 10-day period of August. In 2025, the maximum mean catch was 14 males per trap and was recorded during both the first 10-day period of July and the second 10-day period of August.

Thus, several periods of increased pheromone-trap catches of *Ostrinia* spp. were observed during each growing season, although the timing and magnitude of peak catches varied among years. The highest mean catch recorded over the entire monitoring period occurred during the third 10-day period of June 2024. These interannual differences are presented descriptively and are not interpreted as statistically significant.

No adult moths were captured in the parallel traps baited with *O. nubilalis* pheromone dispensers during the monitoring period. Because attraction to a pheromone dispenser alone is not considered sufficient for species-level identification, these data are interpreted as representing the seasonal dynamics of *Ostrinia* spp. rather than as confirmation of the presence of *O. furnacalis*.

### 3.6. Oviposition and Development of Ostrinia spp.

**Figure 9:**
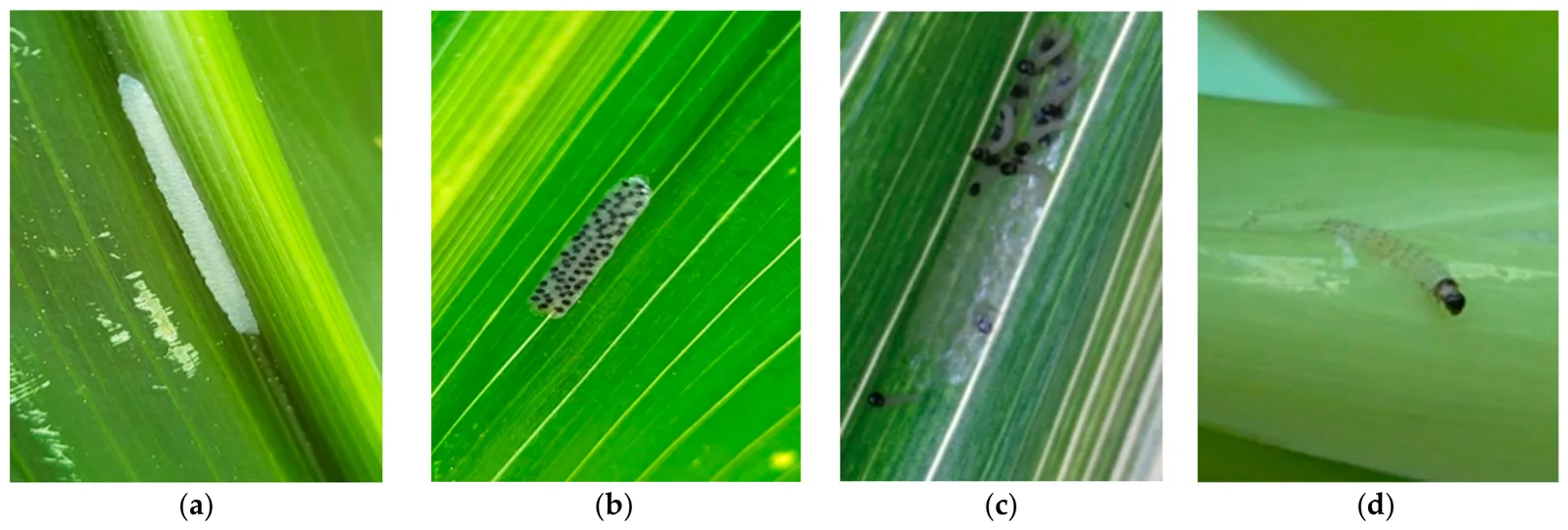
Sequential stages of *Ostrinia* spp. egg-mass development on maize plants: (**a**) freshly deposited egg mass; (**b**) egg mass prior to larval emergence (“black-head stage”); (**c**) larval emergence; and (**d**) first-instar larva after hatching.

The first egg masses of *Ostrinia* spp. were recorded in late May to early June, when maize plants were at the 4–6 true-leaf stage. Egg masses continued to be observed throughout June and, to a lesser extent, into early July, indicating a prolonged period of oviposition activity in the surveyed fields. Egg masses were found predominantly on the abaxial surfaces of leaves located in the middle portion of the plants. The eggs were oval and slightly flattened and were deposited in overlapping groups forming characteristic shingle-like egg masses (Figure 9).

During surveys of 100 marked plants within each sampling plot, egg masses of *Ostrinia* spp. were recorded throughout the observation period, although their occurrence on the examined plants was uneven. Repeated observations allowed the development of egg masses to be followed from the freshly laid stage through the “black-head stage” and subsequent larval hatching. After hatching, the larvae were initially observed on leaf surfaces; at later stages, they penetrated the stalk tissues and continued their development within the plants. In parallel with pest monitoring, naturally occurring parasitoids and predators associated with maize agroecosystems were also recorded during visual surveys.

### 3.7. Assemblage of Naturally Occurring Parasitoids and Predators in Maize Fields

**Figure 10:**
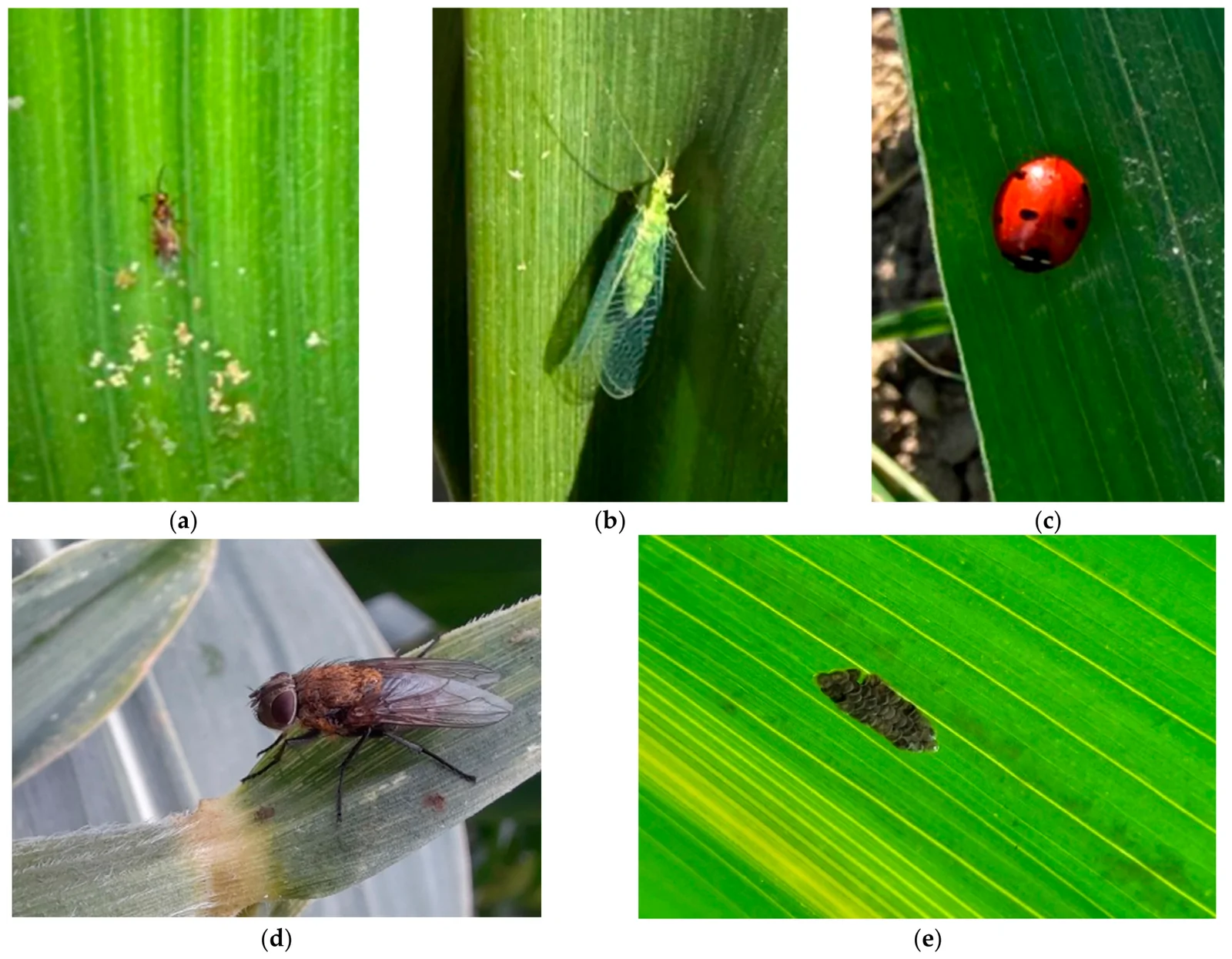
Naturally occurring parasitoids and predators recorded in maize fields of southeastern Kazakhstan: (**a**) representative of Braconidae; (**b**) representative of Chrysopidae; (**c**) representative of Coccinellidae; (**d**) representative of Tachinidae; (**e**) *Trichogramma* spp.

Phytosanitary monitoring of commercial maize fields in southeastern Kazakhstan revealed, in addition to the principal phytophagous insects, an assemblage of naturally occurring parasitoids and predators represented by Braconidae, Trichogrammatidae, Chrysopidae, Coccinellidae, and Tachinidae (Figure 10). The occurrence of parasitoids of the genus *Trichogramma* was confirmed by the collection of naturally laid *Ostrinia* spp. egg masses showing evidence of parasitism and the subsequent emergence of parasitoids under laboratory conditions. Braconidae were recorded from parasitized larvae of lepidopteran pests, with parasitism confirmed by subsequent parasitoid emergence in the laboratory. Chrysopidae and Coccinellidae were observed directly on maize plants, whereas Tachinidae were recorded both during field surveys and during examination of post-harvest maize residues containing overwintering insects.

The obtained results confirm the presence of naturally occurring parasitoids and predators in the surveyed maize agroecosystems. However, differences in the methods used to record these groups do not allow direct comparison of their relative abundance or assessment of their contribution to the regulation of phytophagous pest populations.

**Figure 11:**
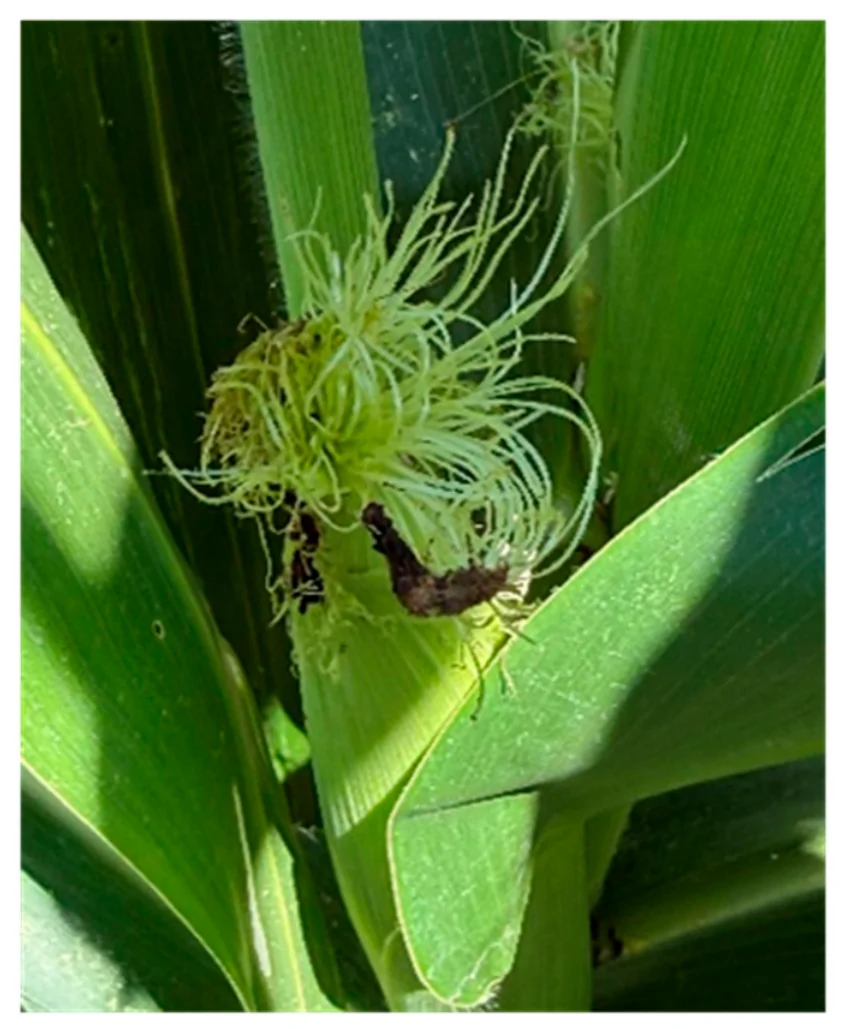
*Ostrinia* spp. larva showing external signs consistent with bacterial infection.

In addition to parasitoids and predators, dead larvae of *Ostrinia* spp. showing external signs consistent with bacterial infection were recorded during the examination of *Ostrinia* spp. specimens. These individuals were collected for subsequent isolation of bacterial strains and their molecular identification (Figure 11).

Six bacterial isolates were obtained from dead *Ostrinia* spp. larvae showing external signs consistent with bacterial infection. These isolates were subjected to molecular identification based on *gyrB* gene sequence analysis.

### 3.8. Molecular Identification of Bacterial Isolates

Based on the analysis of gyrB gene sequences, all six bacterial isolates examined were identified as Bacillus thuringiensis. The obtained sequences showed 99.53–100% similarity to reference sequences of B. thuringiensis available in the NCBI GenBank database using the BLAST algorithm. The detection of B. thuringiensis in the examined material confirms the presence of this bacterium; however, it does not establish a causal relationship between its presence and the mortality of *Ostrinia* spp. larvae, since the pathogenicity of the isolated strains was not experimentally evaluated in the present study.

## 4. Discussion

The present study showed that *Ostrinia* spp. and *Helicoverpa armigera* were the principal lepidopteran pests recorded in the surveyed maize agroecosystems of southeastern Kazakhstan. Pheromone monitoring conducted from 2022 to 2025 demonstrated the occurrence of both taxa during the growing season and revealed interannual variation in the timing and magnitude of seasonal trap catches. These findings are consistent with observations from other regions of Asia, where *Ostrinia* spp. are recognized as important pests of maize and their phenology is strongly influenced by temperature and geographic location [16,17,22].

During the four-year monitoring period, several periods of increased pheromone-trap catches of *Ostrinia* spp. were recorded within individual growing seasons, primarily during June–July and August. The timing and magnitude of these increases varied among years. The highest mean catches recorded during the entire study occurred in 2024, reaching 23 males per trap during the third 10-day period of June and 21 males per trap during the first 10-day period of August. These patterns are interpreted descriptively as periods of increased adult activity and do not provide statistical evidence of a bimodal seasonal pattern or a specific number of generations. Seasonal variation in the activity of *Ostrinia* moths has also been reported in other parts of their geographic range. Studies conducted in China have demonstrated an extended period of adult activity from May to September, with the timing of seasonal peaks varying among years and geographic regions [16,23]. Temperature-dependent development and geographic variation in the life cycle of *Ostrinia* have also been documented by [22]. Thus, the interannual variation in the timing of pheromone-trap catches observed in southeastern Kazakhstan is consistent with previously reported variability in the seasonal activity of *Ostrinia* spp. However, the environmental factors responsible for these differences in the study region were not specifically evaluated in the present study.

Four years of pheromone monitoring of *H. armigera* also revealed interannual variation in both the timing and magnitude of trap catches. Four years of pheromone monitoring of *H. armigera* revealed observed year-to-year differences in both the timing and magnitude of trap catches. The maximum mean catches recorded within individual growing seasons were 18 males per trap in 2022, 14 in 2023, 19 in 2024, and 23 in 2025. The annual maxima occurred during the third 10-day period of July in 2022, the third 10-day period of August in 2023, and the first 10-day period of August in both 2024 and 2025. These patterns are presented descriptively and are not interpreted as statistically significant differences among years [5]. Long-term monitoring in maize fields in Romania similarly demonstrated substantial interannual variation in the abundance and timing of peak pheromone-trap catches of *H. armigera* [6]. Comparable findings have also been reported from maize agroecosystems in Korea, where pheromone monitoring of several lepidopteran pests documented the seasonal occurrence of *H. armigera* throughout the growing period [24,25]. Taken together, the present results indicate that the timing and magnitude of peak *H. armigera* trap catches can vary considerably among growing seasons in southeastern Kazakhstan, an aspect that should be taken into account when designing phytosanitary monitoring programs.

The detection of viable *Ostrinia* spp. larvae in maize crop residues in early spring confirms the persistence of overwintering individuals under the climatic conditions of southeastern Kazakhstan. Overwintering as mature larvae within crop residues is a well-documented feature of the life cycle of *O. furnacalis*, although diapause survival and cold tolerance vary substantially according to geographic origin and thermal conditions. Because the species identity of the local *Ostrinia* population has not yet been conclusively established, comparisons with *O. furnacalis* should be interpreted cautiously. Along with viable larvae, the examination of crop residues revealed larvae parasitized by Braconidae, tachinid pupae, and dead larvae showing signs consistent with bacterial or fungal infection. These observations confirm the presence of parasitoids and individuals showing signs of possible microbial infection in overwintered material; however, the available data do not allow direct estimation of their contribution to mortality or population regulation of overwintering *Ostrinia* spp.

Naturally occurring representatives of Braconidae, Trichogrammatidae, Chrysopidae, Coccinellidae, and Tachinidae were also recorded during the growing season in the surveyed maize agroecosystems. Because different groups of natural enemies were detected using different sampling approaches, the present data confirm their occurrence but do not allow direct comparison of their relative abundance or identification of dominant groups. Of particular interest was the occurrence of egg parasitoids of the genus Trichogramma, as representatives of this genus are widely used in biological control programs targeting *Ostrinia* spp. The potential of *T. ostriniae* for the biological control of *Ostrinia* species has been demonstrated in both laboratory and field studies conducted in China and other parts of Asia [25]. Moreover, long-term studies have indicated the potential of *T. ostriniae* for incorporation into biological control programs targeting *Ostrinia* spp. [15].

In this context, the naturally occurring parasitoids recorded in southeastern Kazakhstan represent promising candidates for further investigation of their potential role in biological control of maize pests. In particular, *Trichogramma* spp. and representatives of Braconidae detected in the surveyed agroecosystems warrant further taxonomic and ecological study. Their potential integration with pheromone-based pest monitoring may represent a useful direction for the development of more environmentally oriented maize protection strategies in the region. However, assessment of their regulatory potential will require additional studies addressing natural parasitism rates, seasonal dynamics, species composition, host associations, and the field efficacy of individual natural-enemy species.

## 5. Conclusions

This study characterized the phytosanitary status of maize agroecosystems in southeastern Kazakhstan and the seasonal dynamics of their major lepidopteran pests. *Ostrinia* spp. and *Helicoverpa armigera* were the principal lepidopteran pests recorded in the surveyed fields, and pheromone monitoring conducted from 2022 to 2025 revealed interannual variation in the timing and magnitude of seasonal trap catches.

Examination of maize crop residues confirmed the presence of viable overwintering *Ostrinia* spp. larvae and also revealed parasitized larvae, tachinid pupae, and individuals showing signs consistent with bacterial or fungal infection. Naturally occurring representatives of Braconidae, Trichogrammatidae, Chrysopidae, Coccinellidae, and Tachinidae were recorded in the maize agroecosystems; however, the available data do not allow direct estimation of their contribution to the regulation of phytophagous pest populations.

Overall, these findings provide a regional baseline for improving phytosanitary monitoring of maize pests in southeastern Kazakhstan and for further investigation of naturally occurring enemies as potential components of environmentally oriented and integrated pest management strategies for maize production in the region.

## Data Availability

The pheromone-trap data supporting the seasonal monitoring results are provided in Supplementary Table S1, including individual catches from the three pheromone traps for each 10-day sampling period. Additional data supporting the findings of this study are available from the corresponding author upon reasonable request.

## Supplementary Materials

The following supporting information can be downloaded at: https://www.mdpi.com/article/10.3390/insects17101033/s1, Table S1: Raw pheromone-trap catches of *Helicoverpa armigera* and *Ostrinia* spp. in maize fields of southeastern Kazakhstan during 2022–2025.
