# Supplementary material for: Phytosanitary Monitoring of Major Lepidopteran Pest and Their Natural Enemies in Maize Agroecosystems of Southeastern Kazakhstan
Source: Insects. 2026 Oct 9;17(10):1033. doi: 10.3390/insects17101033 (PMC13705255; doi:10.3390/insects17101033)
Supplement: Supplementary file 1 [file insects-17-01033-s001.zip › insects-4566288-supplementary.pdf]

Table S1. Raw pheromone-trap catches of *Helicoverpa armigera* and *Ostrinia* spp. in maize fields of southeastern Kazakhstan during 2022–2025.

Individual values represent the number of males captured in each of three pheromone traps during each 10-day sampling period. Mean and SE were calculated from the three traps (n = 3).

Table S1A. *Helicoverpa armigera*

Year	Month	Ten-day period	Trap 1	Trap 2	Trap 3	Mean	SE
2022	May	I	2	2	2	2.00	0.00
2022	May	II	4	2	3	3.00	0.58
2022	May	III	2	6	3	3.67	1.20
2022	June	I	1	1	1	1.00	0.00
2022	June	II	9	3	6	6.00	1.73
2022	June	III	9	11	4	8.00	2.08
2022	July	I	10	12	11	11.00	0.58
2022	July	II	2	3	1	2.00	0.58
2022	July	III	21	19	14	18.00	2.08
2022	August	I	22	12	11	15.00	3.51
2022	August	II	15	12	9	12.00	1.73
2022	August	III	2	3	1	2.00	0.58
2022	September	I	14	8	2	8.00	3.46
2022	September	II	9	16	14	13.00	2.08
2022	September	III	8	3	1	4.00	2.08
2023	May	I	2	3	7	4.00	1.53
2023	May	II	7	3	2	4.00	1.53
2023	May	III	12	6	9	9.00	1.73
2023	June	I	8	7	9	8.00	0.58
2023	June	II	9	7	2	6.00	2.08
2023	June	III	3	7	2	4.00	1.53
2023	July	I	12	11	10	11.00	0.58
2023	July	II	6	13	8	9.00	2.08
2023	July	III	7	8	3	6.00	1.53

2023	August	I	8	5	2	5.00	1.73
2023	August	II	12	2	10	8.00	3.06
2023	August	III	19	12	11	14.00	2.52
2023	September	I	9	10	8	9.00	0.58
2023	September	II	8	8	8	8.00	0.00
2023	September	III	9	5	4	6.00	1.53
2024	May	I	1	1	1	1.00	0.00
2024	May	II	3	3	0	2.00	1.00
2024	May	III	9	5	7	7.00	1.15
2024	June	I	6	7	5	6.00	0.58
2024	June	II	1	10	4	5.00	2.65
2024	June	III	0	7	2	3.00	2.08
2024	July	I	7	11	9	9.00	1.15
2024	July	II	3	9	6	6.00	1.73
2024	July	III	10	23	6	13.00	5.13
2024	August	I	21	19	17	19.00	1.15
2024	August	II	11	14	23	16.00	3.61
2024	August	III	9	16	8	11.00	2.52
2024	September	I	7	0	8	5.00	2.52
2024	September	II	21	9	12	14.00	3.61
2024	September	III	17	2	8	9.00	4.36
2025	May	I	4	8	3	5.00	1.53
2025	May	II	9	10	5	8.00	1.53
2025	May	III	18	13	8	13.00	2.89
2025	June	I	15	7	5	9.00	3.06
2025	June	II	3	3	3	3.00	0.00
2025	June	III	9	4	2	5.00	2.08
2025	July	I	9	11	13	11.00	1.15
2025	July	II	13	8	9	10.00	1.53

2025	July	III	29	17	8	18.00	6.08
2025	August	I	36	14	19	23.00	6.66
2025	August	II	13	15	14	14.00	0.58
2025	August	III	8	17	11	12.00	2.65
2025	September	I	7	12	5	8.00	2.08
2025	September	II	16	8	6	10.00	3.06
2025	September	III	7	6	2	5.00	1.53

Note: I, II, and III indicate the first, second, and third 10-day periods of each month, respectively. Trap 1, Trap 2, and Trap 3 represent individual pheromone-trap replicates. Mean represents the average number of *Helicoverpa armigera* males captured per trap during each 10-day sampling period (n = 3 traps), and SE represents the standard error of the mean calculated from the three trap replicates.

Table S1B. *Ostrinia* spp.

**Year**	**Month**	**Ten-day period**	**Trap 1**	**Trap 2**	**Trap 3**	**Mean**	**SE**
2022	May	II	1	0	2	1.00	0.58
2022	May	III	1	0	2	1.00	0.58
2022	June	I	4	6	5	5.00	0.58
2022	June	II	13	16	10	13.00	1.73
2022	June	III	5	7	3	5.00	1.15
2022	July	I	2	3	1	2.00	0.58
2022	July	II	2	2	2	2.00	0.00
2022	July	III	5	6	7	6.00	0.58
2022	August	I	12	19	14	15.00	2.08
2022	August	II	9	9	6	8.00	1.00
2022	August	III	11	8	11	10.00	1.00
2022	September	I	6	4	5	5.00	0.58
2022	September	II	2	1	0	1.00	0.58

2022	September	III	0	0	0	0.00	0.00
2023	May	II	0	0	0	0.00	0.00
2023	May	III	2	3	1	2.00	0.58
2023	June	I	8	9	7	8.00	0.58
2023	June	II	9	9	12	10.00	1.00
2023	June	III	7	8	12	9.00	1.53
2023	July	I	9	13	14	12.00	1.53
2023	July	II	5	4	6	5.00	0.58
2023	July	III	2	1	0	1.00	0.58
2023	August	I	3	4	2	3.00	0.58
2023	August	II	7	13	16	12.00	2.65
2023	August	III	3	2	1	2.00	0.58
2023	September	I	1	1	1	1.00	0.00
2023	September	II	0	1	2	1.00	0.58
2023	September	III	0	1	2	1.00	0.58
2024	May	II	3	2	1	2.00	0.58
2024	May	III	8	9	7	8.00	0.58
2024	June	I	7	5	3	5.00	1.15
2024	June	II	17	21	13	17.00	2.31
2024	June	III	28	26	15	23.00	4.04
2024	July	I	13	6	2	7.00	3.21
2024	July	II	9	8	10	9.00	0.58
2024	July	III	9	6	12	9.00	1.73
2024	August	I	22	27	14	21.00	3.79
2024	August	II	16	12	17	15.00	1.53
2024	August	III	5	4	6	5.00	0.58
2024	September	I	4	2	3	3.00	0.58
2024	September	II	3	1	2	2.00	0.58

2024	September	III	7	5	6	6.00	0.58
2025	May	II	0	0	0	0.00	0.00
2025	May	III	1	2	0	1.00	0.58
2025	June	I	3	5	1	3.00	1.15
2025	June	II	5	6	4	5.00	0.58
2025	June	III	12	11	13	12.00	0.58
2025	July	I	18	14	10	14.00	2.31
2025	July	II	6	7	8	7.00	0.58
2025	July	III	4	3	5	4.00	0.58
2025	August	I	13	10	7	10.00	1.73
2025	August	II	15	16	11	14.00	1.53
2025	August	III	6	12	9	9.00	1.73
2025	September	I	0	4	2	2.00	1.15
2025	September	II	0	2	4	2.00	1.15
2025	September	III	0	0	3	1.00	1.00

Note: I, II, and III indicate the first, second, and third 10-day periods of each month, respectively. Trap 1, Trap 2, and Trap 3 represent individual pheromone-trap replicates. Mean represents the average number of *Ostrinia* spp. males captured per trap during each 10-day sampling period (n = 3 traps), and SE represents the standard error of the mean calculated from the three trap replicates. Catches were obtained using traps baited with *O. furnacalis*-specific pheromone lures. Pheromone-lure response was used to characterize seasonal flight activity and was not considered sufficient for species-level identification.
